# Anti‐interleukin‐17A and anti‐interleukin‐23 antibodies may be effective against Alzheimer's disease: Role of neutrophils in the pathogenesis

**DOI:** 10.1002/brb3.1504

**Published:** 2019-12-17

**Authors:** Hiroshi Katayama

**Affiliations:** ^1^ Katayama Dermatology Clinic Maebashi Japan

**Keywords:** amyloid β, anti‐IL‐17A antibody, bone marrow, formylpeptide receptor agonist, neutrophil

## Abstract

**Introduction:**

Despite the remarkable progress achieved in the research on Alzheimer's disease (AD), its exact pathogenesis is not fully understood and effective therapies do not currently exist. In order to find effective therapy for AD, I ranged extensively over the literature and found an important paper by Tiffany and colleagues.

**Results and Conclusion:**

Neuroinflammation has been proposed as a possible cause or driving force of AD. The discovery by Tiffany et al. that amyloid β (Aβ) is a formylpeptide receptor 2 agonist indicated that Aβ is a potent chemoattractant for phagocytic leukocytes. Therefore, in all likelihood Aβ attracts peripheral blood neutrophils, monocytes, as well as microglia cells in brain parenchyma, and activates them. However, the role of microglia cells and their precursor monocytes in AD pathogenesis remains elusive. Recently, neutrophils were found to be present in areas with Aβ deposits in AD brain and in transgenic AD model mice. Because brain is vulnerable to the effects of reactive oxygen species (ROS) and neutrophils secrete a large amount of ROS, neutrophils look like a driving force of AD. Therefore, a possibility arises that anti‐IL‐17A and anti‐IL‐23 antibodies are effective against AD, because these antibodies can be thought to interfere with neutrophil trafficking from the bone marrow to the blood circulation and thus inhibit neutrophil infiltration into AD brain. Clinical studies using anti‐IL‐17A and anti‐IL‐23 antibodies in patients with AD are required.

## Graphical abstract

IL‐17A mediates neutrophil migration from the bone marrow. Anti‐IL‐17A antibody inhibits it.

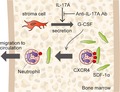

## 1 INTRODUCTION

Alzheimer's disease (AD), a neurodegenerative disease involving loss of cognitive function and memory, affects more than 35 million people worldwide (Querfurth & LaFerla, 2010). However, despite the remarkable progress achieved in AD research (Sanabria‐Castro, Alvarado‐Echeverria, & Monge‐Bonilla, 2017), its exact pathogenesis is not fully understood and effective therapies for AD do not currently exist. During my research on the pathogenesis of psoriasis and the therapeutic mechanisms of anti‐interleukin‐17A (anti‐IL‐17A) (Appendix) and anti‐interleukin‐23 (anti‐IL‐23) antibodies on psoriasis (Katayama, 2018), I considered whether these antibodies would also be effective against AD.

## 2 EMERGENCE OF AMYLOID‐β (Aβ)

Pathologically, AD is characterized by Aβ plaques, neurofibrillary tangles, in the advanced stage, neuronal loss in the neocortex and hippocampus. Aβ is a 36–43 amino acid peptide produced via sequential cleavage of amyloid precursor protein (APP), a transmembrane protein, by the enzymes β‐ and γ‐secretase. Aβ monomers polymerize first into soluble oligomers and then into larger insoluble fibrils, which precipitate in the brain parenchyma as Aβ plaques (Haass, 2004). Neurofibrillary tangles are deposits in the neuronal body of tau, an abnormally phosphorylated microtubule‐associated protein that interferes with cell function.

## 3 NEUROINFLAMMATION IN AD

Although Aβ is directly toxic to cultured neurons in vitro (Mattson, 1997), neuroinflammation has also been proposed as a possible cause or driving force of AD (Wyss‐Coray, 2006). Studies have reported elevated levels of inflammatory mediators in postmortem brains of patients with AD (Heppner, Ransohoff, & Becher, 2015). In the neuroinflammation hypothesis, activated microglia cells are considered key players in AD progression (Block, Zecca, & Hong, 2007; Hoeijmakers, Heinen, Dam, Lucassen, & Korosi, 2016), because microglia cells appear capable of producing superoxide (Shimohama et al., 2000) and various cytokines and chemokines, including IL‐1, IL‐6, TNFα, TGFβ1, TGFβ2, MIP1α, and MCP1 (Akiyama et al., 2000). However, another hypothesis states that microglia cells eliminate amyloid deposits using a cell‐specific phagocytic mechanism (Simard, Soulet, Gowing, Julien, & Rivest, 2006). Therefore, whether microglial activation is detrimental or beneficial for patients with AD remains elusive (Daria et al., 2017; Wyss‐Coray, 2006). By clinical experiments, using ^11^C‐(R)PK11195 and ^11^C‐PIB positron emission tomography and magnetic resonance imaging scans, Fan, Brooks, Okello, and Edison (2017) hypothesized that in the initial phase of AD, microglia cells try to repair neuronal damage, but in the later phase, they become ineffective and produce proinflammatory cytokines, leading to progressive neuronal damage.

## 4 Aβ IS A CHEMOATTRACTANT FOR PHAGOCYTIC LEUKOCYTES

Tiffany et al. (2001) discovered that Aβ is a formyl peptide receptor 2 (FPR2) agonist. FPRs are largely responsible for the detection of invading bacteria, guiding phagocytes to the site of infection, and initiating a cascade of bactericidal activities (Bufe et al., 2015). Briefly, formyl peptides are potent chemoattractants for phagocytic leukocytes (Dalpiaz et al., 2003; He, Troksa, Caltabiano, Pardo, & Ye, 2014; Le et al., 2002). Formyl peptides elicit robust, FPR‐dependent calcium mobilization in human and mouse leukocytes and trigger a range of classical innate defense mechanisms, such as reactive oxygen species (ROS) production, metalloprotease release, and chemotaxis (Bufe et al., 2015). Currently, three functional FPRs have been reported in humans and mice: FPR1, FPR2 (FPR like1, FPRL1), and FPR3 (FPRL2) (Gallo et al., 2014; Liberles et al., 2009). In humans, FPR1 and FPR2 are expressed on both neutrophils and monocytes. The results obtained by Tiffany et al. indicate that Aβ is a potent chemoattractant and activator of phagocytic leukocytes. In addition, it was reported that in the experiment using microfluidic chemotaxis platform, Aβ (soluble form) continuously attracted human microglial cells for 90 hr (Cho et al., 2013).

## 5 NEUTROPHILS AS A CAUSATIVE FACTOR FOR AD DEVELOPMENT

Neutrophils have not been a primary research subject of AD. However, the presence of neutrophils in AD brain was shown by several investigators. Savage et al. (1994) detected cells expressing cathepsin G within AD brain parenchyma and cerebral blood vessels, often associated with Aβ deposits. Zenaro et al. (2015) identified myeloperoxidase^+^ cells in areas with Aβ deposits. Cationic antimicrobial protein 37 (CAP37), which is constitutively expressed in the azurophil granules of neutrophils, was shown in cerebral microcirculation (Grammas, 2000) and in temporal and parietal lobes as well as hippocampal neurons in AD patients (Brock et al., 2015). Studies using 2‐photon microscopy revealed substantial neutrophil migration toward Aβ plaques in transgenic AD mice brain model (Baik et al., 2014; Zenaro et al., 2015). Furthermore, Zenaro et al. (2015) demonstrated that neutrophil depletion or neutrophil trafficking inhibition via LFA‐1 blockade reduced AD‐like neuropathology, in addition to improving memory in mice already displaying cognitive dysfunction.

Once neutrophils migrate toward Aβ plaques, they are activated and secrete harmful mediators, including ROS. Because of its high demand for oxygen and the abundance of highly peroxidizable substrates (Zhao & Zhao, 2013), brain is vulnerable to the effects of ROS. According to Gandhi and Abramov (Gandhi & Abramov, 2012), brain of patients with AD shows evidence of ROS‐mediated injury; there is an increase in malondialdehyde, 4‐hydroxynonenal, and hydroxylated guanine levels in the brain and cerebrospinal fluid of AD patients. Protein carbonyl moieties are increased in the frontal and parietal cortices, and hippocampus in AD brain, with sparing of the cerebellum where no AD pathology occurs (Smith, Richey Harris, Sayre, Beckman, & Perry, 1997).

Another mechanism by which neutrophils damage AD brain is NETosis. Chemokines or ROS initiates a signaling cascade in neutrophils that leads to the disintegration of nuclear and cellular membranes and the formation of extracellular traps (ETs). Zenaro et al. (2015) and Pietronigro, Della Bianca, Zenaro, and Constantin (2017) observed intravascular and intraparenchymal NETosis in the mouse model of AD, potentially harming blood–brain barrier and neural cells. Taken together, these data suggest the involvement of neutrophils in AD progression.

## 6 ANTI‐IL‐17A AND ANTI‐IL‐23 ANTIBODIES AS THERAPEUTIC AGENTS FOR AD

One good therapy may be to use anti‐IL‐17A antibody, because this antibody can be thought to interfere with neutrophil infiltration in psoriasis (Katayama, 2018). Actually, it has been demonstrated that anti‐IL‐17A antibody inhibits neutrophil infiltration into various lesions of animal model diseases, including collagen‐induced arthritis (Kelchtermans et al., 2009), allergic rhinitis (Gu, Wang, & Cao, 2017), asthma, and airway hyperresponsiveness (Ferretti, Bonneau, Dubois, Jones, & Trifilieff, 2003; Hellings et al., 2003; Mizutani, Goshima, Nabe, & Yoshino, 2012), and late‐term corneal allorejection (Yin, Zobell, Jarosz, & Stuart, 2015). In psoriasis, Reich et al. (2015) administered secukinumab, an IL‐17A‐selective human immunoglobulin monoclonal antibody, to 100 patients with moderate to severe psoriasis. This caused rapid improvement of clinical signs and histopathological findings, the most prominent finding being a decrease in the number of neutrophils infiltrating the psoriatic skin. By week 2, the authors found that epidermal neutrophil microabscesses (known as spongiform pustules of Kogoj and Munro's microabscesses) had almost entirely cleared and the dermal neutrophil infiltrate significantly reduced.

**Figure 1:**
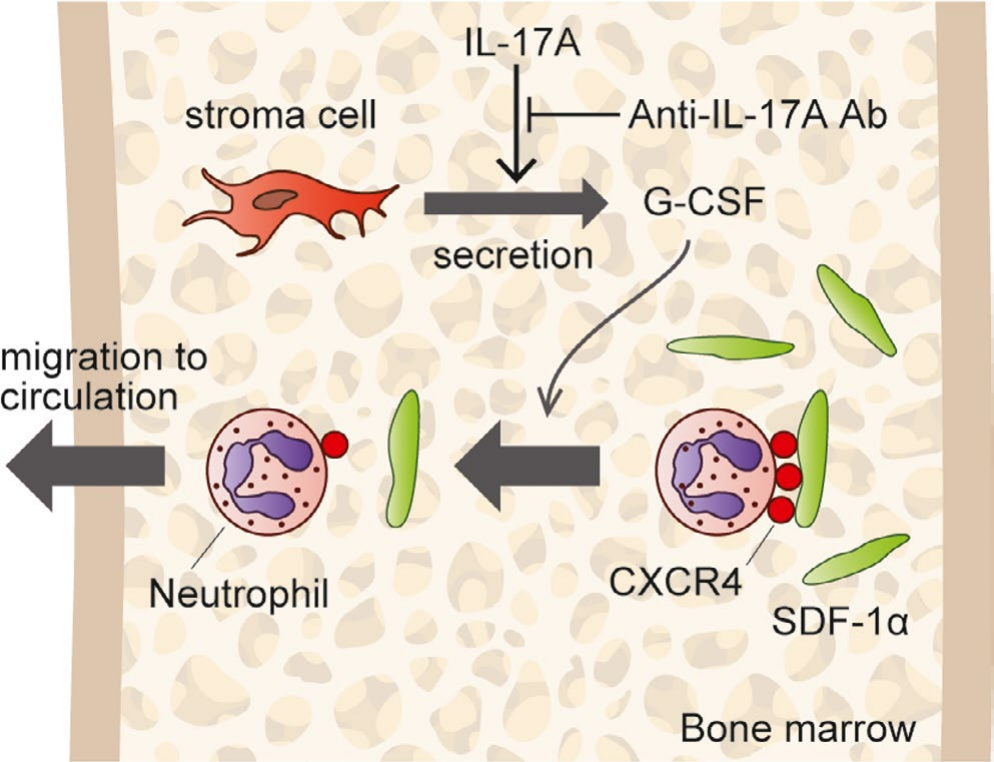
IL‐17A stimulates bone marrow (BM) stroma cells to secrete G‐CSF, and G‐CSF mediates granulopoiesis. Neutrophils in the BM are attached to stroma cell‐derived factor‐1α (SDF‐1α), which is present in BM stroma or on the surfaces of osteoblasts, reticular cells, and endothelial cells. The attachment is mediated by CXCR4 on neutrophil surfaces. G‐CSF downregulates CXCR4 expression on neutrophils and reduces SDF‐1α level in the BM, disrupting the attachment. Neutrophils released from SDF‐1α migrate into peripheral circulation to keep neutrophil homeostasis. Anti‐IL‐17A antibody interferes with IL‐17A‐mediated granulopoiesis and neutrophil migration from the BM and presumably interrupt neutrophil infiltration into AD brain

The inhibitory action by anti‐IL‐17A antibody on neutrophil infiltration, in my thought, is as follows: IL‐17A mediates granulopoiesis, at least in part via granulocyte colony‐stimulating factor (G‐CSF) (Forlow et al., 2001; Kolls & Linden, 2004; Stark et al., 2005), which is secreted from bone marrow stroma cells under the stimulation with IL‐17A (Schwarzenberger et al., 2000). G‐CSF interferes with the transition of neutrophils from viable cells to apoptotic cells (Raam, Drewniak, Groenewold, den Berg, & Kuijpers, 2008; Ramakrishna & Cantin, 2018). Therefore, anti‐IL‐17A antibody drives neutrophils toward apoptosis through G‐CSF deficiency. Another possible mechanism (Figure 1) is a decrease in neutrophil trafficking from the bone marrow to the blood circulation; G‐CSF is an essential regulator of this neutrophil trafficking (Semerad, Liu, Gregory, Stumpf, & Link, 2002). Because neutrophils express CXCR4, a chemokine stromal cell‐derived factor (SDF‐1α) receptor, SDF‐1α appears to act as a retention factor for neutrophils in the bone marrow. G‐CSF reduces the SDF‐1α level in the bone marrow (Furze & Rankin, 2008; Levesque, Hendy, Takamatsu, Simmons, & Bendall, 2003) and also downregulates CXCR4 expression by myeloid lineage cells (Kim, De La Luz, Williams, Gulino, & Tosato, 2006; Levesque et al., 2003). Therefore, G‐CSF deficiency induced by anti‐IL‐17A antibody increases the SDF‐1α level and interferes with neutrophil trafficking from the bone marrow to the blood circulation, inhibiting neutrophil infiltration into various lesions. Anti‐IL‐23 antibody may also induce G‐CSF deficiency, because IL‐17A production by neutrophils depends on IL‐23 and IL‐6 (Taylor et al., 2014). Therefore, anti‐IL‐23 antibody is supposed to act in a similar fashion as anti‐IL‐17A antibody. Involvement of the “IL‐23‐IL‐17A‐G‐CSF axis” in the regulation of granulopoiesis was confirmed in several independent murine models (Wirths, Bugl, & Kopp, 2014). Additionally, anti‐IL‐17A antibody may also interfere with neutrophil recruitment to AD brain through inhibition of IL‐8 production by neutrophils, because IL‐17A, which is produced by neutrophils (Keijsers, Joosten, Erp, Koenen, & Kerkhof, 2014), stimulates various cells, including microvascular endothelial cells, to produce IL‐8 (Linden, 2001; Roussel et al., 2010).

Taken together, these data described above suggest that anti‐IL‐17A and anti‐IL‐23 antibodies, if administered to patients with AD, interfere with neutrophil infiltration into AD brain and inhibit AD progression.

A concern regarding administering anti‐IL‐17A and anti‐IL‐23 antibodies could be neutropenia. However, it seems that clinical trials on patients with psoriasis have not reported serious neutropenia. Probably, in the broad sense of emergency granulopoiesis occurs to maintain neutrophil homeostasis, and this compensates the neutropenia induced by these antibodies. Emergency granulopoiesis is a G‐CSF‐independent process. Several models of neutropenia have shown IL‐17A‐independent feedback regulation of granulopoiesis, implicating redundancy in granulopoiesis‐stimulating signals (Wirths et al., 2014).

Clinical studies using anti‐IL‐17A and anti‐IL‐23 antibodies in patients with AD are required.

## CONFLICT OF INTEREST

The author has no conflict of interests to declare.

## Data Availability

All data generated or analyzed during this study are included in this published article.

## APPENDIX 1

In this study, I used “IL‐17A” instead of “IL‐17,” because IL‐17A is the prototype family member and the use of “IL‐17” is justified to identify “IL‐17A” by the literature.
